# The impact of the National Science Foundation’s Innovation Corps (I-Corps) on academic innovation and entrepreneurship

**DOI:** 10.1140/epjd/s10053-022-00562-9

**Published:** 2022-12-02

**Authors:** Ivy Schultz, John A. Blaho, Kurt H. Becker

**Affiliations:** 1 Industrial Engineering and Operations Research, Columbia University, New York, NY 10015 USA; 2 Innovation and Applied Research, City University of New York, New York, NY 10019 USA; 3 Institute for Invention, Innovation, and Entrepreneurship, Tandon School of Engineering, New York University, Brooklyn, NY 11201 USA

## Abstract

**Abstract:**

In 2011, the U.S. National Science Foundation created the Innovation Corps (I-Corps) program in an effort to explore ways to translate the results of the academic research the agency has funded into new products, processes, devices, or services and move them to the marketplace. The agency established a 3-tier structure to support the implementation of the I-Corps concept. Selected I-Corps teams consisting of the principal investigator, an entrepreneurial lead, and an industry mentor participate in a 7-week accelerated version of the Lean Launchpad methodology that was first developed by Steve Blank at Stanford University. Participating teams engage in talking to potential customers, partners, and competitors and address the challenges and the uncertainty of creating successful ventures. I-Corps sites were set up to promote selected aspects of innovation and entrepreneurship ecosystems at the grantee institutions. I-Corps Regional Nodes were charged with recruiting I-Corps teams in a larger geographical area as well as stimulating a new culture of academic entrepreneurship in the institutions in their area of influence. This Topical Review describes the experiences and the impact of the New York City Regional Innovation Node, which is led by the City University of New York, in partnership with New York University and Columbia University.

**Graphic abstract:**

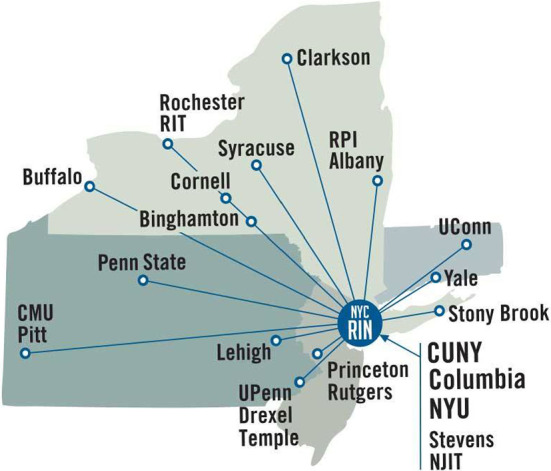

## Introduction

This Topical Review aims to assess and analyze the lasting impact that the New York City Regional I-Corps Node (NYCRIN) had on the innovation and academic entrepreneurship ecosystems at the 3 participating universities, the City University of New York (CUNY), New York University (NYU), and Columbia University (CU), but also how NYCRIN more broadly helped shape the approach to academic entrepreneurship at other universities within the node’s geographical sphere of influence that ranges from Pennsylvania to New Jersey to Upstate New York and Connecticut. The paper starts by reviewing some general aspects of the mission of research universities and attributes of technology transfer in academia. A more detailed analysis of these aspects was recently published [1, 2]. However, we consider it necessary to reiterate below—in an abbreviated fashion—some key observations described in these publications to provide some context for what is discussed in this paper.

For much of the twentieth century, research universities had primarily two functions, (1) teaching, i.e. explaining and passing existing knowledge on to the next generation and (2) carrying out research, i.e. seeking to generate new insights, understanding, and knowledge. In the latter part of the twentieth century, universities were beginning to explore ways to translate research breakthroughs into new products, processes, devices, and services for the benefit of society, but also considered technology transfer an important additional source of revenue [1]. The Bayh–Dole act of 1980 [3], which gave universities the right to control the intellectual property (IP) derived from federally funded research, was a game changer. Universities created patent committees, technology transfer offices, and started to accumulate significant portfolios of IP, especially patents. Incentives for faculty, Postdocs, and students to consider how their research breakthroughs may lead to protectable IP were implemented. Supportive institutional environments and mechanisms to stimulate innovation and academic entrepreneurship were established. To date, there is hardly any major research university in the world that does not claim to be innovative and/or entrepreneurial.

It was also pointed out [1, 2] that technology transfer has largely followed 3 pathways, (1) selling the IP to a third party, often an entity that buys patents in bulk, (2) identifying a licensee to license the IP, or (3) engaging with a partner to jointly develop the technology further toward commercialization. More recently, the creation of spin-out companies has become another path. Spin-out companies license the IP from the university and develop the technology, often with the active participation of the academics who were involved in creating the IP. The last 20 years or so have seen an increasing trend toward start-up formation as an attractive alternative to IP commercialization at many universities. It is undisputed that the failure rate for start-ups is high, by some estimates above 80% [4]. However, if a start-up succeeds, the return on investment can be significantly higher than what can be realized from the more traditional commercialization routes for both the university and the academic inventors.

The U.S. National Science Foundation (NSF) [5] is the premier agency that supports fundamental research and education in science and engineering. NSF's role results in new knowledge and tools as well as a capable, innovative, and educated workforce. Over the past 10 years, the NSF annual budget has increased from about $7b (2011) to $8.8b (2022). In 2010, the U.S. Congress directed the NSF to explore ways to turn the scientific breakthroughs that result from basic research the agency funds into technological advances and increase the economic impact of the research. This led to the creation of the Innovation Corps or I-Corps program [6]. I-Corps is a fast-paced and rigorous program that challenges teams to quickly find out whether or not their ideas and concepts are worth pursuing. The program aims to create a nationwide ecosystem that helps researchers translate their promising technologies to market by teaching them how to be entrepreneurs and connecting them to each other and to potential supporters through a National Innovation Network.

**Fig. 1:**
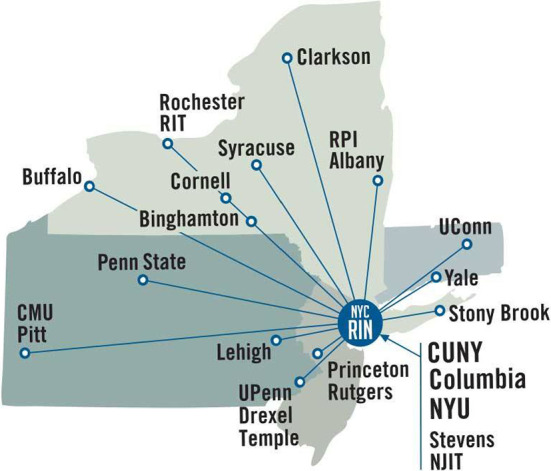
The geographical area of impact of NYCRIN

NYCRIN, the New York City Regional Innovation Node [7], led by the City University of New York (CUNY) in partnership with New York University (NYU) and Columbia University (CU) was among the first I-Corps Nodes [8]. Other universities within the node’s geographical sphere of influence that ranges from Pennsylvania to New Jersey to Connecticut include Carnegie Melon University, the University of Pittsburgh, Penn State University, Lehigh University, Drexel University, Temple University, Princeton University, Rutgers, the New Jersey Institute of Technology, Stevens Institute of Technology, Stony Brook University, the State Universities of New York at, respectively, Buffalo, Binghamton, and Albany, Cornell University, Rochester Institute of Technology, Syracuse University, Rensselaer Polytechnic Institute, Clarkson University, Yale University, and the University of Connecticut at Storrs (see Fig. 1).

## The NSF I-CORPS program

In 2011, the NSF launched the Innovation Corps (I-Corps) program to translate the results of the academic research funded by the agency into new products, processes, devices, or services and move them to the marketplace [6]. The goal of the I-Corps program has been and will continue to help reduce the time and risk associated with translating promising ideas and emerging technologies from the research laboratory to the marketplace. The I-Corps program is designed to primarily support the commercialization of so-called deep technologies, or those revolving around fundamental discoveries in science and engineering. The I-Corps program addresses the skill and knowledge gap associated with the translation of basic research into "deep tech ventures". I-Corps uses experiential learning of customer and industry discovery, coupled with first-hand investigation of industrial processes, to quickly assess the translational potential of inventions.

The heart and soul of the I-Corps program are the I-Corps teams. Each team consists of 3 members, the technical lead, the entrepreneurial lead and an industry (or other external) mentor. The technical lead tends to be the principal investigator or a co-investigator of the research grant that generated the IP. Participating teams, often identified by I-Corps Regional Nodes or I-Corps Sites (see below), need to apply to the NSF and be selected to receive a $50,000 grant in support of their participation. Selected teams are grouped into cohorts of approximately 20 to 30 teams. All 3 team members must participate in a rigorous 7-week curriculum that is an abbreviated version of the Lean Launchpad methodology developed by Steve Blanks at Stanford University [9, 10]. It provides real-world, hands-on, immersive learning about what it takes to successfully transfer knowledge into products and processes that benefit society such as customer discovery (aka getting out of the building/lab), maximizing product-market fit, and completing the so-called business model canvas [9].

In the program’s initial phase, I-Corps Nodes and Sites were established and funded separately to serve as the backbone of the National Innovation Network. I-Corps Nodes were consortia of multiple universities with the responsibility for delivering a standardized curriculum and to impact the academic entrepreneurship ecosystem in universities within their geographical area of influence. NYCRIN, the New York City Regional Innovation Node, was initially funded for 5 years and subsequently renewed for a 2nd 5-year funding period. It ceased operation in August of 2022. Through I-Corps Sites, additional funding was provided to universities to support targeted innovation and entrepreneurship initiatives specific to the scientists and engineers at a given institution and for a more narrowly defined purpose. These complementary building blocks of innovation have led to revolutionary technological advances and entire new industries.

A few years later, the I-Corps program was also introduced at the National Institutes of Health (NIH) [11] and the Department of Energy (DOE) and was expanded to seek the commercialization of research breakthroughs supported by these two funding agencies. NYCRIN leaders played significant roles in the creation and delivery of I-Corps at NIH [12]. In parallel, NYCRIN offered regional I-Corps programs to teams associated with the universities in its network seeking to commercialize IP that was not based on NSF-funded research.

According to the NSF I-Corps 2021 Annual Report [13], since inception, the program has educated more than 5700 individuals and 1900 teams nationwide (all data here are as of the end of the federal fiscal year 2020). These 1900 teams have created about 1000 start-up companies that have raised $760 million dollars in subsequent dilutive and non-dilutive funding. At the May 2022 National Innovation Network (NIN) meeting, VentureWell updated these I-Corps impact numbers to 2523 teams creating 1377 start-ups that raised $1.5B. These companies support 4834 employees [14]. The economic development implication is, by extrapolation, that approximately half of the teams trained at NYCRIN have the potential to form a company that could employ 3–5 deep technology innovators.

In 2017, the American Innovation and Competitiveness Act (AICA, Public Law 114–329, Sec. 601) [15] formally authorized and directed the expansion of the NSF I-Corps program. NSF created the Hubs program to support that expansion. Through it, NSF seeks to evolve the current structure, in which I-Corps Teams, Nodes, and Sites were funded through separate solicitations, toward a more integrated model capable of sustained operation at the scope and scale required to support the expansion of the NSF I-Corps program. In this more integrated model, I-Corps Hubs form the backbone of the National Innovation Network and extend the network to other institutions. CUNY, with its core partners NYU and CU and several other geographically close collaborating institutions, was the recent recipient of a I-Corps Hub award [16].

## The impact of NYCRIN on the participating institutions

Many start-up hubs are located in the proximity of major research universities in the USA. Similar observations are true in other parts of the world, e.g. in Berlin, Munich, Paris, Prague, Innsbruck, London as well as in China, Korea, and India, among others. The reasons for these symbiotic relationships are (1) the strong basic science activities that serve as the foundation of new inventions and innovations, (2) broad expertise in all fields of engineering, applied science and technology, (3) a well-educated and trained human resource base (faculty, students, Postdocs, etc.), and (4) the presence of major tech companies in the area. This is reflected in the location of the prominent NSF I-Corps Regional Nodes. NYCRIN, centered in New York City, has a wide geographical impact region covering research universities in several states bordering New York State as stated above. Other nodes are located in Silicon Valley, the Los Angeles area, the Mid-Atlantic Region, one node centered around Georgia Tech, the Boston area, Texas, the Mid-West region, and Upstate New York.

Even though the basic concept, mandate, and programmatic requirements and expectations are the same for every I-Corps Regional Node, each node realizes them somewhat differently, taking into account that innovation and entrepreneurship ecosystems neither exist in isolation nor are generic, but are an integral part of the local environment, which differs from region to region and even from university to university in the same geographical region.

### Impact of NYCRIN on CUNY

#### Overview

**Fig. 2:**
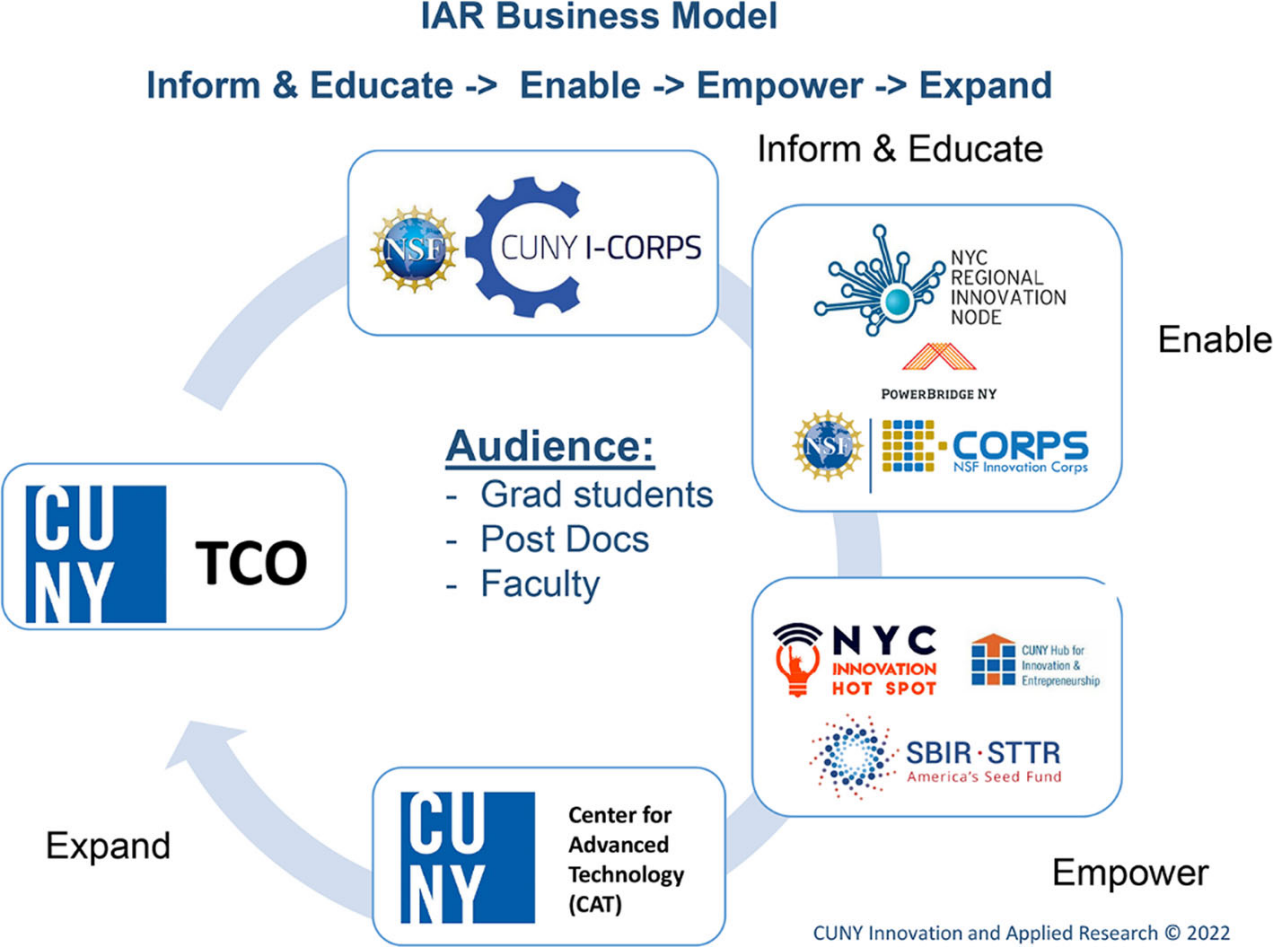
The CUNY IAR Business Model

Due to NYCRIN, the I-Corps process has become the fundamental innovation catalyst for researchers at all levels of CUNY from community college students to graduate students and Postdocs. All I-Corps activities at CUNY have been run since inception under the auspices of the Innovation and Applied Research (IAR) division of the CUNY Office of the Vice Chancellor for Research. The business plan of CUNY IAR is a three-step process. (1) CUNY faculty and students are trained in I-Corps through IAR, (2) they then disclose their IP to the TCO for patent submission, and finally (3) they use the CUNY Hub for Innovation and Entrepreneurship as their business address to submit federal SBIR/STTR grant applications and for continued business development (Fig. 2).

This CUNY IAR business model leads to new research funding, new CUNY start-up company formation, new industry-sponsored research by the new CUNY start-up companies back into CUNY research laboratories, and this leads to the generation of new intellectual property (IP). A major unanticipated outcome is that deep tech researchers who previously did not disclose discoveries to the university are attracted to I-Corps and, upon training completion, then present a portfolio of new use cases—this is a two-pronged win. Increases in technology transfer deals and IP licensing is the largest significant impact that the I-Corps program has had at CUNY.

As a result of new IAR paradigm, I-Corps has become the main driver of new IP generation at CUNY. Each year the CUNY Technology Commercialization Office (TCO) receives approximately 50 new invention disclosures. Between 2013 and 2016, CUNY was awarded approximately 20 patents each year. In 2018, CUNY had 48 disclosures but had 37 patents awarded and this increase has continued through 2022. The Director of the CUNY TCO states that this major expansion in patent issue rate is directly the result of higher-quality patent submissions by CUNY I-Corps innovators. In fact, I-Corps is directly responsible for CUNY reaching the top 100 world-wide universities in patents issued. By August 2022, CUNY had 43 researchers serve a PIs and win $50,000 NSF I-Corps Teams awards, bringing over $2M of new research funding into research laboratories at every one of the 25 colleges at the university.

#### CUNY undergraduate engineering I-Corps

The founding concept of NSF I-Corps was that science and engineering graduate students and their faculty adviser partner with industrial mentors to “pressure test” whether their research innovations might have a commercial potential. At the time (2012–2013), only engineering schools with graduate programs were early adopters of I-Corps. These early evangelists became internal advocates at their institutions who went on to create the original deep technology incubators and maker spaces in the country. Numerous NYC deans of engineering schools reached out to CUNY I-Corps to inquire as to whether we might be able to develop programming for undergraduate students as well. The original pilots were run at the Zahn Innovation Center at the Grove School of Engineering of the City College of CUNY. At this time, the Cooper Union had just hired a new engineering dean who wished to go one step further and require that EVERY freshman engineering student take our I-Corps course. Finally, the new I-Corps Site at the University of Pennsylvania that NYCRIN had helped create wished to extent our course to not only include engineers but business and medical school students. The challenges that had to be overcome in each of these scenarios and the significant lessons that were learned have been presented at national conferences and have been well-documented [17, 18].

#### Masters in translational medicine (CUNY translational medicine I-Corps)

The success of the CUNY Engineering I-Corps process did not go unnoticed. When the Grove School of Engineering hired a new internationally recognized biochemical engineer as its new dean, they reached out to CUNY I-Corps to assist them in creating a new research Master’s degree program that would use I-Corps as the center of its training concept. The dean was able to secure initial funding support for the endeavor from Andrew S. Grove, CUNY Engineering class of 1962 and cofounder of Intel Corporation. CUNY I-Corps partnered with a consultant whose industry career was in the medical device sector. The engineering I-Corps curriculum described above was submitted to the NYS Board of Regents as part of the course accreditation process. The result of this collaboration was the creation of a novel research-based, graduate-level, translational medicine (MTM) program [19]. The MTM program was broken into three segments that ran over a 12-month period. It begins in the fall semester with identification of an unmet medical need and extends to cover intellectual property freedom to operate, and an initial analysis of predicate regulatory approvals. Students form virtual companies and work in teams such that by the end of the semester they have a first design concept and an initial company business model canvas.

The 2^nd^ phase occurs in the spring when the students participate in the Translational Medicine I-Corps process [20]. The teams are further broken into new business units or divisions based on the various indications they develop through an ideation process. Each division takes the design through the I-Corps customer discovery process, while they are simultaneously working in the bio-design laboratory to create prototypes based on customer feedback. In the final stage, the students work full time during the summer to optimize their prototypes and, if they are fortunate, they may test their products in a clinical setting. Important outcomes from the CUNY Translational Medicine I-Corps process are that students have gone on to create companies, succeeded in winning $50,000 NSF I-Corps award, and have been accepted in accelerators.

#### PowerBridgeNY (Cleantech I-Corps)

With funding from the New York State Energy Research and Development Authority (NYSERDA), NYU in partnership with CUNY established the clean technology PowerBridge Proof-of-Concept Center (POCC). Simultaneously, NYSERDA funded a similar POCC effort led by Columbia in partnership with Stony Brook, Brookhaven National Laboratory and Cornell Tech New York City. NYSERDA subsequently approved the proposal to merge them to form PowerBridge NY (PBNY) with the aim of accelerating the transition of emerging clean technologies from academia to viable business ventures [21].

NYCRIN and PBNY partnered to create and deliver six cycles of PBNY’s validation program that is based on the I-Corps process. In the summer of 2015, NYCRIN-PBNY delivered its first Clean Tech I-Corps cohort. The defining features of Clean Tech I-Corps is that all cohort teams/companies must argue that their solution fits in the clean tech sector and that all teaching team members have clean tech development experience. Since then, NYCRIN also helped develop and deliver PBNY’s Hacking-for-Energy program in which several CUNY student teams enrolled in a for-credit course offered from Columbia University. The impact at CUNY is that all PBNY-funded teams went on to successfully receive $50,000 NSF I-Corps Team awards and almost all formed start-up companies that went on to win STTR/SBIR funding performing industry-sponsored research back into CUNY research laboratories.

#### Community college innovation challenge and Steve Blank Fireside chats

**Fig. 3:**
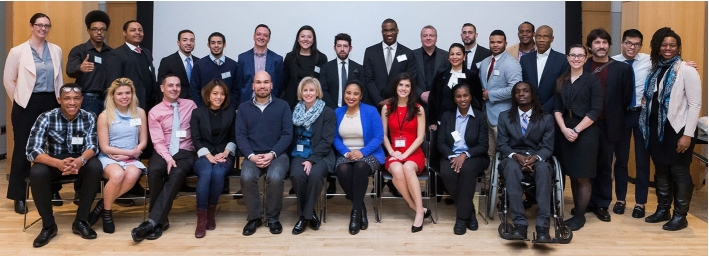
CUNY-Capital One Community College Innovation Challenge Lessons Learned presentation at CUNY Graduate Center in 2017

In 2014, Capital One Bank supported the development of a pilot program for teaching entrepreneurship to Community College students as part of their American Community Reimbursement Act responsibilities. To scale the program to other colleges throughout CUNY it partnered with CUNY IAR to develop a scalable I-Corps based program [22]. The Capital One-CUNY Community College Innovation Challenge was supported by Capital One from 2014 to 2019 (Fig. 3).

The success of the program was not lost on innovation leaders the national level where the NSF copied the program name for themselves [23]. In 2016, the Innovation Challenge formed the basis of the CUNY NSF I-Corps Site that established CUNY I-Corps.

The CUNY I-Corps Site is unusual in that it involves spreading the I-Corps program to our Community College population, as well as to other affiliated institutions. CUNY has a large Community College component whose student population is highly creative and adaptable. CUNY is extremely diverse; 60% of the student population is female, a large proportion is economically challenged, and other typically underrepresented cohorts in STEM (Hispanics, racial minorities, etc.) make up a large portion of the student population. The entrepreneurial culture changes enabled by CUNY I-Corps have been profound for this population. Overall, the CUNY I-Corps Site should serve as a national model for inclusion.

One of the most enjoyable aspects of the Innovation Challenge is that for four consecutive years, CUNY IAR hosted Steve Blank for a Fireside Chat that was moderated by Harry Smith. These chats we organized around the same time period that Steve came to NYC each year to lecture at the Columbia Business School. The first chat was in 2016 and took place at Baruch College of CUNY. Based on the vast turnout for these events, they were moved to the Graduate Center of CUNY. The 2017 event was designed to coincide with the formal lessons learned presentations by the Innovation Challenge students.

**Fig. 4:**
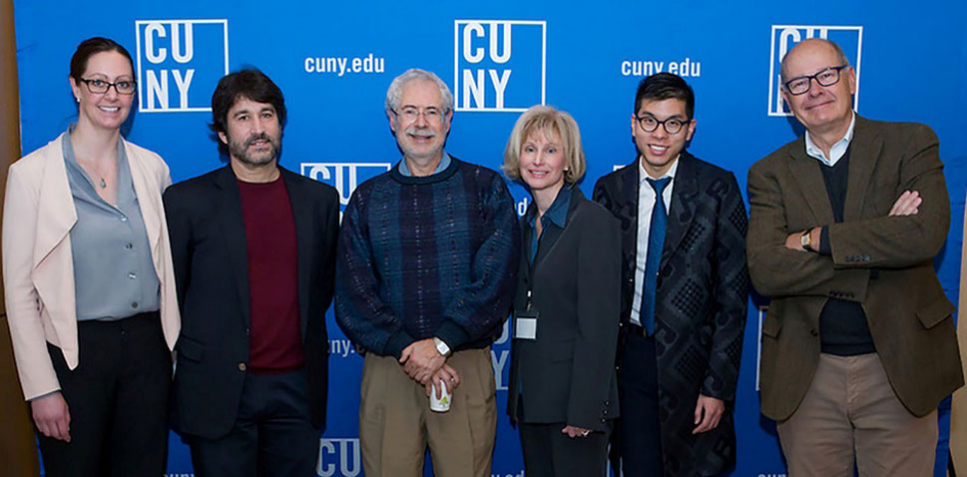
Lydia McClure, John Blaho, Steve Blank, Christine Mooney, Philip Loew, and Harry Smith at the CUNY Graduate Center in 2017

Steve’s title for the chat in 2017 was entitled, “Innovation versus Entrepreneurship:

What’s the Difference and Why Does it Matter?” (Fig. 4) In 2018 at the Graduate Center, Steve and Harry discussed, “Has Silicon Valley Become the Place it Hated? The last chat occurred in 2019 and was designed to acknowledge the fact that Steve Blank was raised in Queens Village, NY. Steve spoke at his alma mater, Martin Van Buren High School on “Looking Back and Looking Forward” [24]. Van Buren has had a partnership with Queensborough Community College (QCC) called Business Technology Early College program (B-Tech) where high school students take credit-bearing courses at CUNY. The impact of this is that Van Buren students have gone on to participate in the Innovation Challenge as they were officially enrolled in QCC classes.

The impact of CUNY I-Corps and the Innovation Challenge is that these university-wide programs created an I-Corps-based innovation and entrepreneurship infrastructure at its predominantly undergraduate colleges. This provided the basis for the Blackstone Group to leverage what was built [25]. The 9 new CUNY Blackstone LaunchPad campuses are home to the most active Innovation Challenge schools, so I-Corps set the stage for Blackstone to come in and seamlessly substitute for Capital One Bank.

#### CUNY Center for Advanced Technology (CUNY CAT) and Industry-University Cooperative Research Centers (IUCRCs)

I-Corps at CUNY evolved out of the university’s traditional entrepreneurial center, the Empire State Development-sponsored Center for Advanced Technology in Photonic Applications, termed the CUNY CAT. In partnership with the CAT, CUNY IAR brought in two new NSF Industry-University Cooperative Research Centers at the City College of CUNY; IUCRC for Metamaterials and IUCRC for Sustainably Integrated Buildings and Sites. IAR currently serves as the innovation and entrepreneurship advisors for CUNY’s recent IUCRC for Building Energy Smart Technologies and IUCRC for Biological Applications of Solid-State Systems. During 2011–2012, the first four CUNY national I-Corps teams were from CUNY CAT laboratories. In the fall of 2012, when the original NYCRIN node application was being prepared, the focus of CUNY IAR pivoted to focus its efforts on I-Corps. By the time the CUNY CAT was renewed in 2018 and reorganized as the CUNY ASRC Sensor CAT, CUNY IAR and CUNY I-Corps became again the CAT’s essential business development pipeline such that by 2022 almost all CAT projects are with I-Corps companies.

#### NYC innovation hot spot (NYC IH)

In 2014, New York State Governor Andrew Cuomo announced the Cycle IV NYS Regional Economic Development Council (REDC) funding award winners in Albany. The CUNY IAR was named the Innovation Hot Spot for the New York City REDC. The New York City Innovation Hot Spot (NYC IH) is an Empire State Development business incubator at the City University of New York (CUNY). Located in the West 125th Street Opportunity Zone corridor in Manhattan—a federal initiative that encourages private investment in low-income urban and rural communities—the NYC IH provides business and workforce development services to the Upper Manhattan and South Bronx and works closely with technology incubators throughout NYC.

The NYC IH provides business incubator services in Harlem and is operated in coordination with CUNY I-Corps. The NYC IH is charged with coordinating the regional entrepreneurial ecosystem and has partnered with over 40 local deep technology incubators encompassing all five Boroughs of NYC. The NYC IH provides opportunities for these incubators and their members to participate in NSF I-Corps Lean Launchpad entrepreneurship training, as well as certain New York State tax benefits. The NYC IH accepts referrals from these incubators for potential Innovation Hot Spot tax benefits, which may ultimately lead to START-UP NY certification at a CUNY location.

Students, faculty, and individuals from the community explore the viability of new ventures through boot camp-style cohorts modeled after the successful National Science Foundation I-Corps Lean Launchpad entrepreneurial training program, which prioritizes customer discovery in an iterative process that is constantly revising the potential product or offering, and culminates with a final “lessons learned” presentation/pitch. This intensive training enables start-up companies to scale into sustainable businesses. The NYC IH also guides these companies through their partnerships and distribution channels to ensure use of an efficient supply chain. Participants take advantage of CUNY’s extensive facilities and equipment resources. Following the cohort, new ventures receive one-on-one mentoring support in securing non-dilutive Small Business Innovation Research/Small Business Technology Transfer grants, as well as seed/early-stage venture capital financing.

#### NYC business plan competition hosted by CUNY I-Corps

In 2021, CUNY I-Corps and the NYC Innovation Hot Spot agreed serve as the regional organizer and host of the NYC Business Plan Competition (NYCPBC). The regional semifinals are hosted by local partner colleges and universities, and the top teams from each of the 10 regions advance to the final round of the state-wide competition. 12 NYC student-led teams are eligible to advance to compete in the State-wide Competition. 16 teams completed at the NYC Regional Business Plan Competition. 12 teams from this regional were nominated to the state-wide competition to compete among 120 teams total. The State-wide Final Competition took place at the Venture NY event, hosted by Upstate Capital, on May, 2021. Seven state-wide Business Plan Competition awards were given to the New York City student-led team winners. Awards given to the seven teams from NYCRIN partner schools included:

- VAMPIRO, Touro College—1st Place in MedTech & Life Sciences Track
- Fiction Stop, NYU—1st Place in Consumer and Business Products Track
- Minority and Women-Owned Business Enterprise Award to NorML, Ichan School of Medicine at Mount Sinai
- Social Entrepreneurship Award—Grandma's House, Touro College
- Venture-Backable Business Award—EZ Health, Columbia University
- Pandemic Response Award—NorML, Icahn School of Medicine at Mount Sinai

#### Gotham Innovation Gambit (GIG innovation Lean Boot Camp)

In 2021, The CUNY iHub and I-Corps programs came together to create the innovation-based economic recovery project entitled the Gotham Innovation Gambit (GIG) that is supported by the Economic Development Administration of the US Department of Commerce. This new program delivers economic development to Upper Manhattan and its surrounding area, contributing to the resurgence of an economically distressed community by meaningfully expanding opportunities for disadvantaged people. The GIG focuses on entrepreneurship support to promote technology-enabled entrepreneurialism and enable access to people and ventures that may not normally pursue IP-based, deep-tech development and deployment.

New ventures are trained in boot camp-like cohorts modeled after the successful NSF I-Corps Lean Launchpad entrepreneurial training program, culminating with a final lesson learned presentation/pitch. This new training program is called the Innovation Lean Boot camp, and it encourages innovators from the community who are not university-affiliated to participate. Following the cohort, new ventures receive one-on-one support in securing non-dilutive SBIR/STTR grants, as well as seed/early stage venture capital financing. Successful completion of this project will serve as a recovery and resilience model to be shared throughout the country. The GIG Lean Boot Camp has emerged to become to predominant I-Corps training paradigm at CUNY.

### Impact of NYCRIN on NYU

#### Overview

Much of what was said before regarding the impact of the I-Corps process on the CUNY innovation and academic entrepreneurship environment is equally applicable to NYU. At NYU, Technology Opportunities and Ventures (TOV) is the gatekeeper for invention disclosures, patent filings, patents granted, licensing, and start-up formation. Since 2013, NYU has consistently been included in the list of the top 100 universities worldwide in terms of parents granted, ranking between 20 and 40 with an average number of patents granted annually ranging between 60 and 90. The number of patents granted is, of course, a lagging indicator of the inventive spirit of faculty and students as it can take more than 3 years from a patent filing to the granting of the patent by the US Patent and Trademark Office (USPTO). Exposure to I-Corps training at NYU has consistently led to an increase in technology disclosures, but, more importantly, it resulted in more mature and more developed technology disclosures. This, in turn, has led to a 40% increase in technology disclosures that ultimately resulted in a patent filing over the past 5 years.

As mentioned before, the commercialization and monetization of faculty-developed and university-owned IP through spin-outs from the university has become an important part of any university’s IP commercialization strategy. NYU is no exception. I-Corps had a profound and tangible impact on the university’s start-up creation. Before I-Corps, many start-ups were created prematurely, which led to a high rate of failure. I-Corps has taught potential founders that patience is a virtue. Engaging in customer discovery, researching product-market fit, developing a concise value proposition, identifying channels to market, strengthening the IP are important components for the creation of a start-up with a higher potential for success. In essence, I-Corps taught founders to delay company creation until they have a much better understanding of customer needs, market acceptance, and have developed a go-to-market strategy. More importantly, as long as no start-up company has been formed, potential founders and their teams can avail themselves of the assistance and the various support mechanisms that the university can provide. Start-up formation creates a for-profit commercial entity. This limits the ability of the university to continue to support the start-up so as to not jeopardize its 501(c)(3) tax-exempt status by selectively providing support to certain commercial entities. While we have no hard numbers yet, anecdotal evidence suggests that start-ups that are formed after going through some of the components of the Lean Launchpad have a significantly rate of success (as measured e.g. by the number of start-ups that are still in business 2 years after company formation).

#### The NYU Entrepreneurial Institute and the Mark and Debra Leslie eLab

Entrepreneurial activities involving faculty, researchers, and students across many Schools and College across NYU started prior to the establishment of NYCRIN. The NYU Entrepreneurial Institute (NYU EI) was established in 2010 [26] as a university-wide entity, administered by the Provost’s Office, with the mission to create a diverse inclusive culture that facilitates and celebrates entrepreneurship campus-wide, supporting students, faculty & researchers creating scalable solutions to meaningful problems. However, the initial offerings were modest, somewhat uncoordinated, not well integrated into the academic mission of the various Schools and Colleges, and lacked broad faculty buy-in. The advent of NYCRIN provided an opportunity for a reassessment and realignment of the NYU EI activities and to establish a new framework. Some offerings were phased out, others were modified with an eye on introducing and integrating elements of the Lean Launchpad methodology, and new offerings were conceived to expand the portfolio of services. Key personnel of the NYU EI served as I-Corps instructors from the beginning and, over the next few years, more individuals from NYU and from entities in the NYU EI network served in that role or as Teaching Assistants or mentors. Given that one mandate of the I-Corps program was to prepare teams for the national I-Corps program, the NYU EI aimed to identify teams with potential and create a sequential pipeline of programs that provide the necessary guidance and support to maximize the team’s chances of being accepted.

These efforts of the NYU EI were greatly aided by the opening of the Mark and Debra Leslie eLab, a 6800 square-foot physical space that started to house the NYU EI and its programs in 2014 and served as a place where faculty, researchers, and students can meet to connect, collaborate, and tap into a vast array resources to help develop their ideas and inventions into start-up companies.

#### The NYU summer Launchpad

The NYU Summer Launchpad (SLP) is the flagship program of the NYU EI. The program, which was launched about a year after NYCRIN was established, is an immersive 9-week program for 8–10 teams of NYU students who want to explore if their idea has market potential. They receive one-on-one coaching with New York City mentors, investors and entrepreneurs, customer development training, legal and accounting services and $10,000 in non-dilutive funding**,** in a dynamic, community-minded atmosphere. Now in its 10th year, the program was the launch pad for many successful NYU start-ups. Alumni of the program have collectively generated more than $200 million in annual revenue, have created over 400 new jobs**,** and have raised over $160 million in venture capital and non-dilutive funding.

#### The NYU start-up accelerator series

In the following years, two programs preceding the Summer Launchpad were conceived in an effort to better prepare the teams entering the Summer Launchpad. This resulted in a 3-level Start-up Accelerator Series that provides sequenced skills-development and support to advance NYU start-up teams to the next level based on evidence-based insight and mentorship. The Series begins with customer discovery in the Start-up Boot Camp, continues with a fast Start-up Sprint, and culminates in the Summer Launchpad. Start-up Boot Camps are two half-day workshops with a week of customer discovery interviews in between for teams who have shown commitment to moving their ideas forward and who have been working on their idea-stage venture for a few months before applying. Accepted teams are taught how to test the value of their ideas and validate (or invalidate) the problem they are solving for their target customer. In the process, teams will refine their technology and may pivot on the road to a more successful start-up based on customer feedback. Start-up Sprints are two-week programs supporting aspiring NYU entrepreneur teams to transform their ideas and inventions into businesses by challenging them to deepen the understanding of their customer base in order to develop successful solutions which meet their needs, i.e. sharpen the "problem–solution fit". Start-up coaches guide the teams through skills-building workshops and approached to grant funding and peer support**.**

#### Inclusion and diversity

**Fig. 5:**
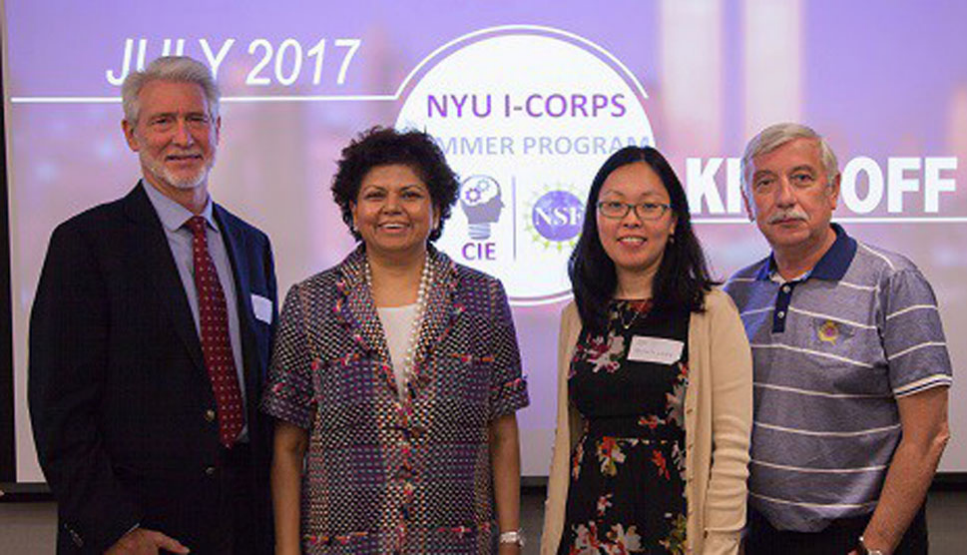
Group photo of Peter Voltz (Associate Dean for Academics), Chandrika Tandon (Chair, NYU Tandon Board of Overseers), Jin Kim Montclare (PI of the NSF Site) and Kurt Becker (Vice Dean for Research, Innovation, and Entrepreneurship)

Fostering inclusion and increasing diversity has been an important component of NYCRIN’s mission. This is also the case at NYU. The NYU Tandon School of Engineering was home to a 5-year I-Corps Site devoted to “Enhancing Diversity in STEM Entrepreneurship” [27] from 2017/18 to 2021/22 (Fig. 5). The objective was to support about 30 entrepreneurship teams per year from all Schools and Colleges of NYU, with an emphasis on enhancing STEP entrepreneurship among women and traditionally underrepresented minorities. Students, faculty advisors, and mentors engaged in an immersive summer program based on the Lean Launchpad methodology infused with insights into enhancing and supporting diversity through e.g. partnering with the NYU Tandon Office of Inclusive Excellence (OIE) The goal of the OIE is to create an academic environment that is reflective of the diversity of our country and our global community by offering strategic leadership for Inclusion, Diversity, Equity and Belonging at Tandon and aims to build and foster diversity, equity, inclusion, social justice, anti- racism, and anti-discrimination. In addition to the immersive summer program, the site also established an accelerated program during the J-term in January before the start of the spring semester. Over the 5-year life of the site, 180 teams successfully completed the program, 113 teams during the summer and 67 teams during the J-term.

Perhaps more important than the numbers of successful program graduates is the subsequent trajectory of many of them. Many went on to participate in other programs and competitions such as PowerBridgeNY, the NYU InnoVention competition, in Tandon’s Vertically Integrated Projects (VIP) Program, the Women in StatrtUp competition, the NYU Stern $300 k competition, the NYU Stern $100 k Entrepreneurship Challenge, the NYU Prototyping Fund, Y Combinator, the Princeton Entrepreneur Competition. Some graduates were admitted into the Tandon Future Labs (see below) and several applied successfully for VentureWell grants and, after company formation, for SBIR/STTR grants. The Site set in motion the creation of a pipeline of teams (and start-ups) that would, in some cases after exposure to additional training and education, participate in the national and regional I-Corps cohorts. Below is a small selection of graduates and their technology, which shows the diversity of technologies that the site has been supporting:

- Team G-ware developed a smart fitbit for the aging with a care coordination system for more comfortable and secure living
- Team Merciless Motors built a vastly improved electric motor, 90% more efficient, 33% more powerful and 20% lighter than current electric motors
- Team “We are the New Farmers” set up an urban farm growing sustainable produce for specialty markets that lack year-round local supply
- Team Dollaride addressed the transportation needs of people who live in areas of NYC that are poorly, if at all serviced by public transportation by setting up a “dollar van” network
- Team Pi-Radio developed a low-power, fully digital transceiver for future communication applications using mm-waves (6G)
- Team CODie provided a user-friendly mobile application that helps students learn basic coding through gamifying the learning experience
- Team Aergen engineered solutions that spread or significantly minimize the spread of air-borne infectious diseases in health care setting and beyond (inspired by the challenge of controlling the spread COVID-19)

In addition, the NYU EI established a Female Founders Fellowship program that provides female founders with access to a robust community of entrepreneurs at NYU who are committed to advancing gender equity in entrepreneurship, as well as extensive training, mentorship, and networking opportunities. Upon graduation, Female Founders Fellows are also invited to apply for grants of up to $50,000.

#### The Veteran entrepreneurship program and the Veterans future lab

**Fig. 6:**
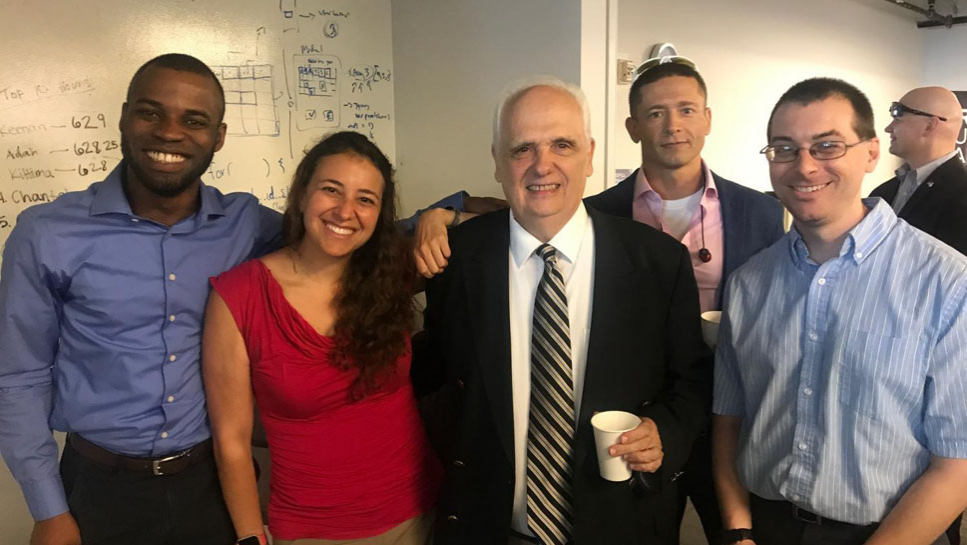
Elana Duffy, founder of Pathfinder Labs, and Assemblyman Joseph Lentol (center) are flanked by several graduates of the 2017 VET program

In 2016, NYU Tandon was approached by NYS Assemblyman Joseph Lentol with a proposal to provide entrepreneurship training to military veterans and their spouses. Assemblyman Lentol, a military veteran himself, argued that many attributes of our servicemen and servicewomen are similar to what makes a successful entrepreneur. In response, the NYU Tandon School of Engineering created an immersive 12-week Veteran Entrepreneurship Training (VET) program consisting of an initial half-day boot camp, followed by 10 weeks of once-a week lectures and hands-on exercises using many components of the I-Corps concept and Lean Launchpad methodology. The VET program culminates in a final demo day (Fig. 6). Since its inception, the program has provided an introduction to entrepreneurship to more than 200 veterans and their spouses. Several graduates of the first few VET cohorts, upon graduation, proceeded to form very early stage start-ups to explore the viability of their technologies, gauge customer interest, and assess market potential. As a result, NYU Tandon created a 1-year incubation program to support these veteran-founded and veteran-led start-ups, the so-called Veterans Future Lab, VFL [28]. The VFL accepts early-stage start-ups and provides them with a curated portfolio of services, a connection to the NYCRIN network, and introductions to a national network of partners, advisors, and mentors, who also support entrepreneurship among military veterans.

#### The NYU Tandon future labs

**Fig. 7:**
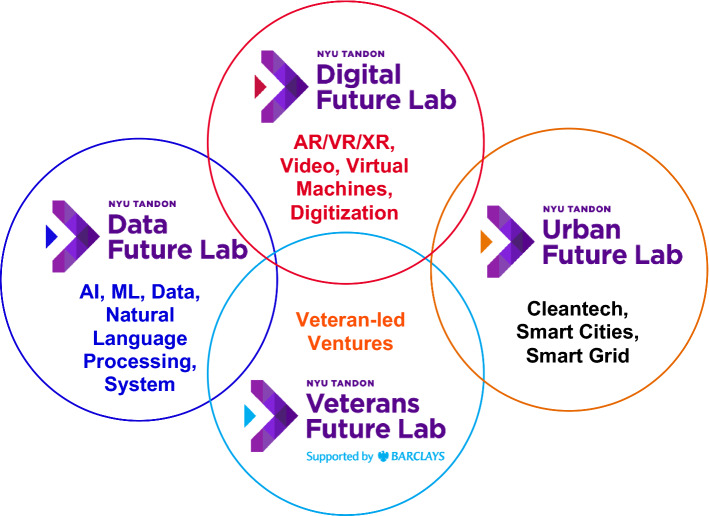
The NYU Tandon Future Lab Ecosystem

What is now the NYU Tandon School of Engineering, started to develop its own innovation and entrepreneurship ecosystem in 2009, when the School was only affiliated with NYU—it did not become part of NYU until 2014. In 2009, the School started two incubators that supported primarily very early-stage start-ups, one of them exclusively dedicated to cleantech start-ups, the other one serving several technology verticals. This network was expanded in subsequent years and the focus also shifted to supporting start-ups between seed stage and series A financing (or equivalent). This resulted in what is now called the NYU Tandon Future Labs (Fig. 7), a network of Technology Acceleration and Commercialization hubs consisting of the Data Future Lab, the Digital Future Lab, the Urban Future lab, and the Veterans Future Lab. The Future Labs offer 2 programs, a 6-month boot camp for early-stage start-ups, called “Catalyst” and a 2-year “Seed to Series A financing (or equivalent)” incubation program.

After the merger with NYU in 2014, the innovation and entrepreneurship ecosystems at NYU and NYU Tandon began to collaborate more closely and combined their resources for the benefit of faculty, Postdocs, and students in all units of NYU. The success of the Future Labs is in no small measure due to the fact that accepted start-ups have access to a broad portfolio of support services and mandatory training that incorporates many components of the Lean Launchpad methodology and also leverages the programmatic support of the NYU EI and of NYCRIN.

Since the Future Labs were created with seed funding from New York City (NYC) and New York State (NYS), they could not limit acceptance of start-ups to only those affiliated with NYU, but were required to open their door initially to start-ups from NYC and NYS. Subsequently, the Future Lab leadership decided to cast an even wider net and accept start-ups for across the United States and later also from foreign countries.

While the Future Labs were initially created at arm’s length from the academic side of the university, they have had an increasingly profound impact over the years on shaping many aspects of the academic mission and curricular offerings. Select faculty serve as Faculty Engineers in Residence in the Future Labs. They provide guidance and expert advice to the start-ups, but, perhaps more importantly, they often use technical challenges faced by start-ups as test cases in their classroom teaching. The Tandon faculty has implemented a program where students can take internships with Future Lab start-ups for academic credit. However, the most important result of aligning the operation of the Future Labs more closely with the academic mission of Tandon has been a gradual infusion of entrepreneurial thinking into the engineering curricula, ranging from modest changes to existing course syllabi to new courses such as the Forum on Innovation, a required course for all Engineering Freshmen or a required course in entrepreneurship as part of a degree program to the spearheading the establishment of an Engineering Entrepreneurship Minor that is offered by the Department of Technology Management and Innovation and open to any engineering major.

#### The Institute for invention, innovation, and entrepreneurship at NYU Tandon

In 2017, NYU Tandon created the Institute for Invention, Innovation, and Entrepreneurship (IIIE) [29] as the focal point of all research, educational, and service activities, as well as extra-curricular activities, in support of the School’s goal to integrate invention, innovation, and entrepreneurship into its academic culture and to advance student and faculty appreciation of and skills in inventiveness, innovation, creativity, and entrepreneurial thinking. The IIIE works closely with the NYU EI, but unlike the NYU EI, the IIIE is much more closely aligned with the School’s academic mission. The IIIE draws its strengths from the contributions of faculty from multiple departments, a structure that facilitates the integration of inventions and innovations emerging from the departmental faculty and students and linking them to entrepreneurship, i.e. the proactive consideration and realization of commercialization opportunities through various institutional pathways. Specifically, the IIIE:

- serves as a one-stop shop for all Tandon-wide activities that promote invention, innovation, and entrepreneurship.
- supports a nurturing environment for the translation of science and engineering breakthroughs into new technologies, products, and processes in service to society.
- assists in the implementation of new learning opportunities that will provide every student with some basic knowledge and experience of entrepreneurial thinking.
- connects faculty and students with the NYU Tandon Future Labs as well as with other innovation and entrepreneurship activities across the entire university.
- promotes student participation in NYU Tandon and other NYU innovation and entrepreneurship competitions.
- links the NYU Tandon community to the broader NYC technology scene through hackathons, workshops, competitions, guest lectures and tech talks.
- creates and supports the infrastructure for faculty and students to turn their ideas into successful ventures
- oversees many student-centric competitions and activities such as the NYU Global InnoVention competition (https://engineering.nyu.edu/research-innovation/entrepreneurship/nyu-global-innovention-competition/innovention), the Tandon Made Challenge (https://engineering.nyu.edu/research-innovation/entrepreneurship/nyu-tandon-made-challenge), the A/X Venture Studio (https://futurelabs.nyc/programs/flax/), and the NYU Translational Healthcare Initiative (https://engineering.nyu.edu/research-innovation/entrepreneurship/nyu-translational-healthcare-initiative).

### Impact of NYCRIN on CU

#### Overview of impact

The impact of the I-Corps process on the CU innovation and academic entrepreneurship environment is quite similar to that on CUNY and NYU, specifically to CU Engineering, where the I-Corps program resides. The I-Corps methodology has impacted both curricular and co-curricular actives for students, faculty, staff, alumni, and our surrounding community. While it impacted certain areas in a very direct way, such as the Translational Fellows Program, it has also shaped our design challenges and residential programs for start-up-curious undergraduate students.

At Columbia, Columbia Technology Ventures (CTV) manages invention disclosures, patent filings and start-up formation for faculty-led innovations. Columbia has consistently been included in the list of the top 20 universities worldwide in terms of parents granted, with a range of 98–119 patents per year between 2013 and 2020. The Columbia Lab-to-Market (L2M) Accelerator Network plays a critical role in facilitating the commercialization of research [30]. The I-Corps experience at Columbia through the many programs and courses has led to an increase in teams attending bootcamps, cash awards, commercial launches, and external funding.

#### The translational fellows program (TFP) at Columbia Engineering

The TFP program was launched in 2016 to support faculty research commercialization through the support of postdoctoral researchers with programming based on the I-Corps methodology. The program supports 20% of the salary of selected postdoctoral researchers and research scientists in the Columbia School of Engineering and Applied Sciences (SEAS) for one year. This is a competitive, nomination-based postdoctoral research program that funds selected fellows for one day per week, providing them with the opportunity to pursue commercialization of a technology that originated in SEAS research. In addition to following the I-Corps customer discovery methodology, the program provides mentoring resources, conflict-of-interest (COI) management, and other support to maximize the opportunity to create a new venture. The TFP aligns resources available through CU’s entrepreneurial ecosystem to accelerate the pace of technology translation. TFP fellows use the business model canvas as a main tool while doing customer discovery to validate the market potential of their technologies.

TFP provides the opportunity to study the commercial opportunity and potential markets of a specific body of IP generated through research, connect with mentors and the CU entrepreneurship ecosystem, assess the necessary technological development and the path to a minimal viable product, and meet advisors, fellow engineer-entrepreneurs, and potential investors to begin fundraising.

#### Start me up boot camp

In order to prepare start-up teams for the future—whether it is a National NSF I-Corps, fundraising, or otherwise launching a business, Columbia Engineering implemented the Start Me Up Boot Camp which runs quarterly. This two-week boot camp guides start-ups through the customer discovery process and the basics of business model development. The program kicks off for two half days, has a mid-point check in, and culminates in a half-day finale. All teams go through an interview process—during this process our faculty and staff consult with teams and help with mentor-matching, should a team need a mentor for the national program. The boot camp is open to teams across the Columbia campus and teams in the NYCRIN community. During the pandemic, the cohorts were held virtually; this led to the current format which is now hybrid. Students attend the kick-off and check in portions of the program online and the finale is in person. Our finale usually consists of a presentation on Start-up Fundraising and Start-up Legal Basics—it is attended by participants, current students, and our alumni community, which allows for networking for the start-up teams.

#### Developing an entrepreneurship ecosystem in Tunisia

CU has frequently partnered with the organization Open Start-up Tunisia (OST), an international non-government organization (NGO) whose objective is support the youth in Tunisia to develop ideas for innovative start-ups that address global challenges. Columbia applied many lessons learned from the National Innovation Network in the USA to the local context in Tunisia. Tunisian start-ups and faculty also visited CU and NYC to learn about the New York ecosystem. Tunisian students are paired with Columbia Business School students who help them through the customer discovery process as they develop their pitches. OST is now in its 5th year; over 30 teams have now been selected and coached through the customer discovery process. These teams were coached by professional trainers, experienced professors, entrepreneurs as well as MBA students from CU. Columbia also facilitates a “Train the Trainers” Program, which includes an introduction to I-Corps and how to leverage the lean methodology, trainings on how to engage professionals from industry to mentor and coach, examples of how to launch products, and simulation activities on the entrepreneurship cycle and process.

Ivy Schultz and Dario Vasquez attended the Open Conference provided by VentureWell in Washington, DC March 28–30, 2019 and presented on “Building the Ecosystem: Tunisia, a Global Success in Scaling Innovation” [31] as part of the Mini Replicating I-Corps Across Regional Ecosystem. They shared best practices for launching programs in a new local context and lessons learned based on experience.

#### Residential Inc. (Res. Inc.) Program

Res. Inc. is Columbia Engineering’s residential program for undergraduate engineering students interested in entrepreneurship. The Res. Inc. program is based on the I-Corps curriculum and provides young students with access to faculty and professionals in their fields of interest, weekly workshops to develop entrepreneurial skills, and the tools necessary to develop a business model canvas for their projects. In the first half of the fall semester, students form product teams and complete an intensive overview of the Lean Start-up curriculum. In the second half of the semester, they focus on developing a pitch and compete for funding against other undergrads, graduate students, faculty, and alumni in the Engineering School’s annual competitions. Res. Inc. teams, composed primarily of first- and second-year students have now graduated and are running successful start-ups around the world.

#### Hacking for energy

Students in the program from the NYCRIN core universities formed teams to address clean-tech stake holders challenges and NYCRIN and PowerBridgeNY leveraged these efforts. Hacking for Energy is a semester-long course designed to engage graduate students toward learning about and helping to solve real-world technological and business problems facing the energy and sustainability sectors, while also exposing them to the lean approach to entrepreneurship. Modelled after Steve Blank’s *Hacking for Defense* class (regularly taught at Stanford) and several other universities), Hacking for Energy features problem statements supplied by Industry Hosts for energy and sustainability issues that need immediate solutions. Student teams will propose a solution to a problem statement and spend 14 weeks determining if it is viable using the lean methodology for customer discovery. Over 4 semesters, graduate students from Columbia, NYU, and CUNY spent 14 weeks developing solutions to real energy and sustainability challenges from large organizations. Each week the student gave updates on their development in class to a set of eight instructors experienced with the methodology. They also received regular guidance from their team mentor as well as their Industry Hosts.

#### Design challenges at Columbia University

Columbia University runs design challenges on topical areas, from the Ebola crisis to the Opioid Epidemic. Design Challenge teams at Columbia University now conduct customer discovery as part of their design process. Design challenges are also incorporated into for-credit courses at the University. For example, during the Census2020 Challenge, student teams visited target communities in New York City (September–December 2019) to speak with local residents, understand the needs of the community, develop potential ideas for designing prototypes to assist in the NYC count for 2020.

Teams focused on understanding the needs of hard-to-count groups such as Hasidic Jews, Chinese-Americans, Chinese Immigrants, and young children (under 5), and working with non-profit groups and libraries to increase response rates. Some teams used open-sourced federal data to develop models to predict 2020 Census response rates. The projected predictions can be used to redirect funding and resources both before and during the Census count to increase response rates in areas that may have otherwise been missed. Future design challenges will continue to use the lean methodology and challenge student to apply human centered design to better understand the needs of those they are serving, whether they are individuals or organizations.

#### Columbia Venture competition and ignition grants

The University-wide Columbia Venture Competition allows students to compete in six different themed tracks for non-dilutive funding. All teams must demonstrate knowledge of their customer-base and the Technology and Start-up Columbia tracks put additional weight on customer discovery learnings. After the competition, students can apply for Ignition Grants as a follow-on application. Student teams who win Ignition Grants are highly encouraged to apply for the customer discovery boot camp and are all required to submit milestone reports that track their progress using the lean methodology among other milestones. The I-Corps methodology has become a significant part of students’ curricular and co-curricular activities on campus and students are now used to the idea that they must do customer interview in order to understand the market potential for their ideas.

### Impact of NYCRIN beyond the Core Participating Institutions

#### Unique use of NYCRIN nationally certified Instructors

**Fig. 8:**
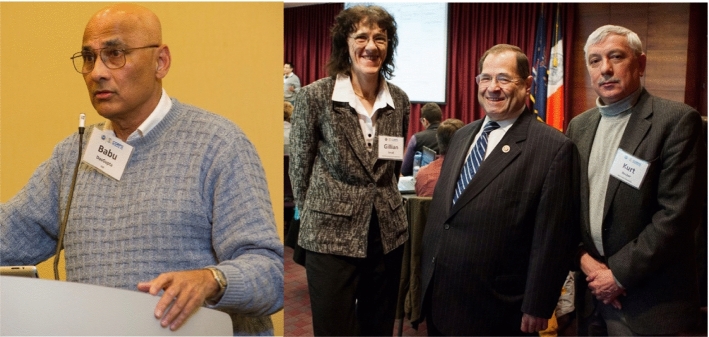
First National I-Corps Cohort April 2013

NYCRIN has 13 nationally certified instructors, of which 46% are women. The instructors have delivered 11 National Cohorts where 230 National I-Corps Teams have been trained. NYCRIN-run national I-Corps cohorts have differed from those of any other Node since its very first cohort in April 2013 (Fig. 8). More national cohort details are given below.

Between 2014 and 2018, NYCRIN delivered full, canonical regional cohorts that were actually one week longer than NSF national cohorts. At the end of 2018, the NSF program director at that time requested that NYCRIN no longer “compete” with the NSF national cohorts and begin offering short regional cohorts. Between 2018 and 2022, NYCRIN ran 34 regional short cohorts, where 374 teams graduated and 1017 participants were trained. It should be noted that these numbers reflect cohorts in which teams from all NYCRIN regional institutions participated and do not include local, institution-specific courses delivered at CU, NYU, and CUNY.

**Fig. 9:**
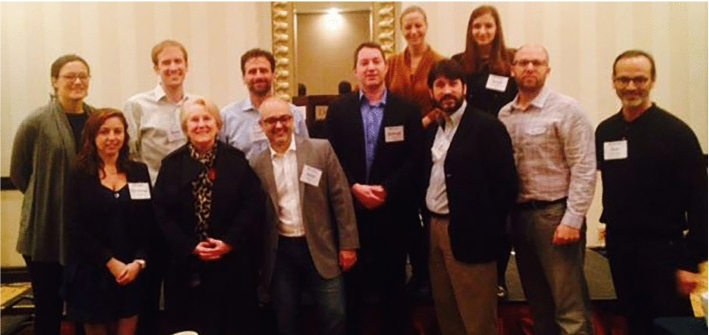
“Ice-Corps” teaching team—EWR 2015

NYCRIN traditionally used national cohorts to train regional economic development leaders in the I-Corps process by allowing them to serve as national adjuncts. While most other Nodes struggle to get the minimum needed six teaching team members, NYCRIN routinely would include six or more adjuncts (Fig. 9). This innovation proved impactful as many NYCRIN adjunct then went on to become PIs of regional I-Corps Sites.

NYCRIN national cohorts have always utilized their own regional teaching teams. Occasionally, the NSF has requested that we include junior instructors from other Nodes who would benefit from additional national cohort experience. The fact that has since 2013 NYCRIN always fielded its own instructor slate represents another example of how NYCRIN remains unique from the other Nodes. In addition, several NYCRIN nationally certified instructors have effectively “graduated” from teaching in NYCRIN national cohorts so that newer instructors may get experience. The graduated NYCRIN national instructor have become a significant part of the national instructor group since they all teach at every other Node. NYCRIN instructors not only teach in NSF I-Corps cohorts. They have been founding and continuing instructors in the NSF SBIR Boot Camps. They are also important members of the I-Corps at NIH and the new NSF SBIR-only I-Corps cohort teaching teams. Thus, no other Node in the national innovation network has impacted the teaching of I-Corps across all disciplines as much NYCRIN has done.

#### NYCRIN annual network meetings—role in creating regional I-Corps Sites

NYCRIN has used its annual network meetings to build its innovation ecosystem. The first network meeting was held in July 2013 at the NYU Law School and the founder of NSF I-Corps, Errol Arkilic, detailed for NYU, CU, and CUNY leadership the rationale and planning that went into creating the new NSF program. Since then, the annual meetings have rotated to CU and CUNY campuses. These meetings have been attended by NSF program directors, academic leaders from all of the regional schools, I-Corps team members, instructors, and mentors, as well as numerous ecosystem stakeholders. The 10^th^ Annual NYC I-Corps Networking meeting was held at the CUNY Advanced Science Research Center in July, 2022 and the current NSF I-Corps program director, Ruth Schuman gave the Keynote address.

**Fig. 10:**
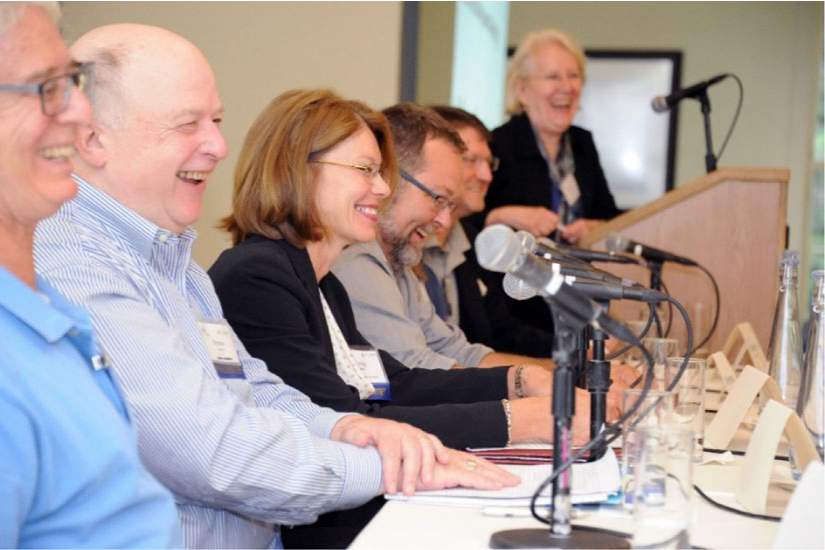
Anita LaSalle (far right) moderating the 2015 Sites panel

One of the most innovative feature of the network meetings was the I-Corps Sites panel. The idea was that when a new Site was awarded in the NYCIN region, the Site lead would describe what their first year activities were. The hope was that after their first year, they could share their lessons learned and best practices. The first Sites panel occurred in 2015 at the Columbia faculty house and it was moderated by Anita LaSalle, the NSF I-Corps Sites program director. The 2015 panel included representative from the UPenn, MIT, CMU, UDel, UConn, and RIT (Fig. 10).

**Fig. 11:**
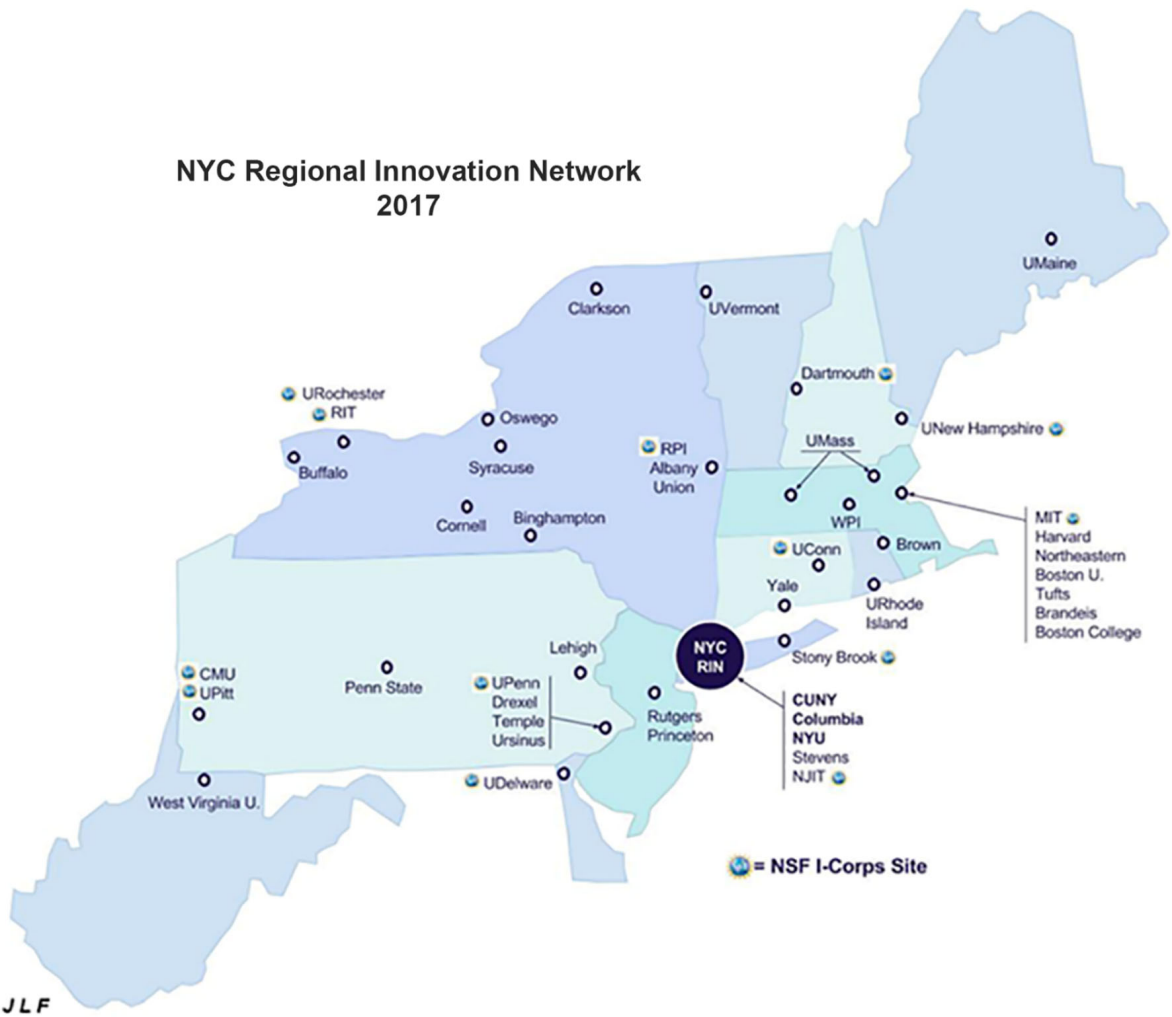
The NYC regional innovation network in 2017

The unexpected impact of the Sites panel at NYCRIN annual meetings was that schools that were interesting in submitting their own Site application had a venue where they could pose questions to and network with the most recent Site award winners in the NYCRIN region. Word quickly spread and university representatives from over 20 different states routinely began attended these meetings. In this manner, the NYCRIN network meetings firmly established its central role in the newly evolving national innovation network. The impact of this is that by 2017, NYCRIN’s field of influence extended from Morgantown, WV, to Orono, ME, where many of the individuals who either were adjuncts in NYCRIN cohorts or attended NYCRIN network meetings were now serving as PIs of new I-Corps Sites. As a result, it became necessary to refer to NYCRIN as meaning the NYC Regional Innovation Network (Fig. 11).

#### I-Corps at NIH and NCATS

In the summer of 2014, Christie Canaria and John Blaho flew out to Steve Blank's ranch and hammered out what became the syllabus for the original I-Corps at NIH. The original pilot launched in the fall of 2014 in Bethesda, MD and several NYCRIN instructors participated in the process**.**

**Fig. 12:**
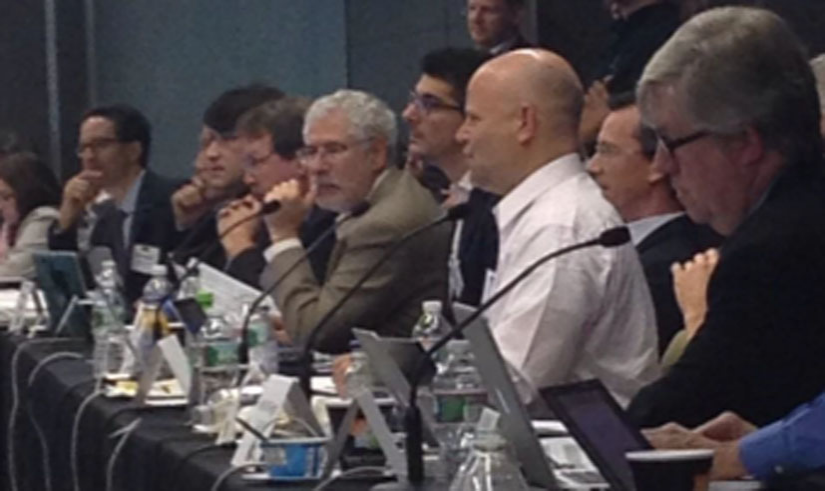
I-Corps at NIH Instructor team in 2015

NYCRIN faculty have continued to serve as sector experts and general I-Corps instructors (Fig. 12) and were involved in the final update of the I-Corps at NIH prior to it going remote during Covid-19.

In 2016, the NIH National Center for Advancing Translational Sciences (NCATS) created a series of Clinical and Translational Science Award program hubs at over 50 institutions throughout the US. Both NYCRIN and the NY I-Corps Hub has worked closely with those NCATS programs at Columbia, Rockefeller, and UMass Medical to assist them in developing their own introduction to I-Corps training programs. High quality teams identified in I-Corps@NCATS have been referred to NYCRIN regional training activities. Today, the NY I-Corps Hub coordinates with the I-Corps@NCATS consortium to source deep technology national I-Corps teams.

#### NSF SBIR phase 0

In 2018, NYCRIN SBIR Phase 0 Teams were drawn from across the country. NYCRIN is proud to say that the Phase 0 program was overall a success. NYCRIN nominated 12 teams to the Spring 2018 cohorts. All were accepted but 1 team backed out due to changes in their schedule. Additionally, 2 NYCRIN teams completed the Mentor cohort in Indianapolis in Spring 2018.

#### NSF I-Corps inclusion summit

In early July 2018, NSF I-Corps leaders Anitia LaSalle and Pam McCauley requested that NYCRIN assist them in running a major networking event in early October 2018. NYCRIN submitted a conference proposal to support this effort. Representation of ALL NSF I-Corps Nodes and Sites was expected and occurred. The main focus was the participation of over 100 "Guests of Honor" who were leaders (Presidents and Provosts) from HBCUs, HSIs, Tribal Colleges, and Schools serving the Deaf. NYCRIN invited and paid the travel and accommodations for all of these guests. The main focus of the meeting was to introduce I-Corps and other relevant NSF programs to the guests. Special effort was made to increase the amount of networking opportunities between the guest and the I-Corps Sites and nodes with the goal of fostering future partnerships.

#### Culturally relevant economic development (CRED)—Tribal Colleges I-Corps

In 2018, a pilot cohort was created to facilitate partnerships between NSF, NYCRIN, and Native America/Alaskan Native (NA/AN) communities in the USA and to develop an innovation and STEM/STEAM-based entrepreneurship curriculum and incubator program. The program was built through the combination of ideas and needs of NA/AN communities together with customer discovery methodologies from the NSF I-Corps program as appropriate. The goals were to create a robust ecosystem of NA/AN entrepreneurship supporters at Tribal Colleges and partnering institutions, document the components of the Business Model Canvas and lean methodology that can meet unmet NA/AN community needs, and to support commercialization of NA/AN deep technology designed to meet the needs of these communities.

**Fig. 13:**
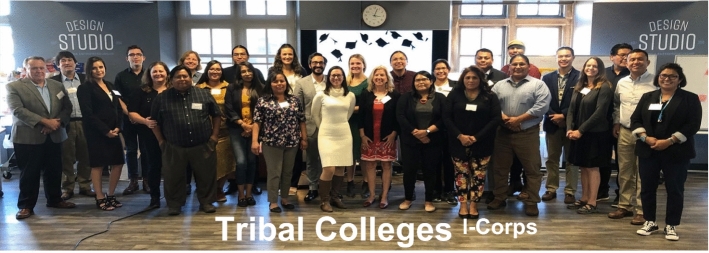
Finale—CU, 2019

Eight teams were recruited from five universities and three tribes to participate in an eight-week course covering the RHS of the BMC. Additional lectures were designed in collaboration with the tribal community input and members including competitive teaching team was led by NYCRIN instructors in collaboration with the LA node, MIT Solve fellows, UN fellows, and tribal leaders. Each team applied with a mentor and was given access to a cohort-wide layer of community mentors sourced from tribal day kick off was hosted in concert with Arizona State I-Corps affiliates in Chandler, entrepreneurial networks, industry leaders, and the NYCRIN and NSF networks. The kickoff occurred in Arizona to accommodate local travel for a majority of the teams. Seven sessions were held via WebEx and the finale was hosted at the Columbia Design lab in New York City in coordination with the MIT Solve/UN Summit to expand the team’s networks (Fig. 13). NYCRIN attended the American Indian Science & Engineering Society conference later that year to disseminate learnings from the pilot, recruit collaborators for future cohorts, and continue to determine how best to evolve the CRED program to support NA/AN entrepreneurs and their communities through the NSF.

#### Development of NC HBCU I-Corps ecosystem

In Fall 2020, NYCRIN worked with leaders from three HBCUs in North Carolina to develop a pilot regional pipeline I-Corps program in accordance with their own NSF EAGER entrepreneurship training grant. The schools were North Carolina A&T State University (NCATSU), North Carolina Central University (NCCU), and Winston-Salem State University (WSSU). The initial course that was developed included eight teams, two of which went on to win $50,000 national I-Corps Team awards. All three HBCU leaders have gone on to participate as volunteer adjuncts in both NYCRIN regional and national I−Corps cohorts. In Spring 2021, they continued their support of the pipeline program being developed with NCA&TSU-NCCU-WSSU, focusing this second phase of the program on training the trainers. NYCRIN continues to work with these leaders as they develop their collective pipeline to the nationals and local programs at each of their universities. The development of this mentoring program has been presented at national innovation meetings and has now been accepted for publication [32].

## Summary

Deep technology teams from New York-region institutions of higher education (IHE) were adopters that participated in the original 2011–2012 national I-Corps cohorts at Stanford University led by Steve Blank. Centered around the blossoming New York City innovation ecosystem, a consortium of 25 leading research IHEs in the greater metropolitan area came together to establish the first competitively awarded NSF I-Corps Node. The NY I-Corps Node fostered the creation of most of the NSF I-Corps Sites in its area. In its latest iteration, the NY I-Corps Hub is now geographically focusing its efforts more deeply into its firmly established local innovation ecosystem. Part of its mission is to include IHEs that have not traditionally participated in I-Corps and to embrace its location at the heart of a large group of major medical institutions.

The City University of New York (CUNY) is the hub's principal institution, with Columbia University and New York University as partner institutions. The hub includes five initial affiliates: Icahn School of Medicine at Mount Sinai, Rockefeller University, Stevens Institute of Technology, Stony Brook University, University of Massachusetts Medical School, and University at Albany. The hub will expand by adding new member institutions each year.

## Declarations

### Data Availability Statement

This manuscript has no associated data or the data will not be deposited. [Authors’ comment: This article did not generate any new data.]
